# Bifokale Perspektive in der Arbeit mit Familien mit psychisch erkrankten Eltern: Training zur Verbesserung von Einstellungen, Wissen und Fertigkeiten für medizinische Fachkräfte

**DOI:** 10.1007/s00278-021-00557-8

**Published:** 2021-12-09

**Authors:** Svenja Taubner, Lea Kasper, Sophie Hauschild, Silke Wiegand-Grefe, Anna Georg

**Affiliations:** Institut für Psychosoziale Prävention, Universitätsklinikum Heidelberg, Bergheimer Str. 54, 69115 Heidelberg, Deutschland

**Keywords:** Fachkräftetraining, Psychisch kranke Menschen, Kinder beeinträchtigter Eltern, Kindeswohl, Psychiatrie, Training for mental health professionals, Mentally ill persons, Children of impaired parents, Child well-being, Psychiatry

## Abstract

**Hintergrund:**

Psychisch erkrankte Eltern stellen einen Risikofaktor für die transgenerationale Weitergabe psychischer Störungen dar. In der Psychiatrie mit ihrem Fokus auf das Individuum werden Patient*innen nicht immer als Eltern erkannt.

**Ziel der Arbeit:**

Entwicklung und Evaluation eines Trainings für medizinische Fachkräfte zur Unterstützung einer Familienorientierung in der Psychiatrie unter der Maßgabe einer bifokalen Perspektive, die den Indexpatienten und die Familie mit Schwerpunkt auf die Kinder im Blick behält, werden vorgestellt.

**Methoden:**

Zur Etablierung der bifokalen Perspektive in Einstellungen, Wissen und Fertigkeiten wurde ein halbtägiges Training, bestehend aus einer Vorlesung und einem Seminar, entwickelt. Dieses wurde im Rahmen des Konsortiums Children of Mentally Ill Parents – Research-Network (CHIMPS-NET) an 7 Standorten in Deutschland in den dortigen Erwachsenen- sowie Kinder- und Jugendpsychiatrien durchgeführt. Der Bedarf wurde vor dem Training per Online-Fragebogen erfasst. Die Implementation wurde durch die qualitative Auswertung von Gedächtnisprotokollen der Trainerinnen begleitet.

**Ergebnisse und Diskussion:**

Das Training konnte erfolgreich mit 120 Teilnehmenden durchgeführt werden, wobei das Ziel einer berufsgruppenübergreifenden Schulung des gesamten Personals, auch pandemiebedingt, nicht realisiert und die übenden Elemente des Trainings nicht gut genutzt werden konnten. Die Auswertung der Fragebogen, die ca. 50 % der Teilnehmenden ausfüllten, ergab bei der Gruppe der teilnehmenden Psychologinnen und Ärztinnen bereits eine deutliche Familienorientierung. Die qualitative Protokollauswertung aller Standorte zeigte den hohen Bedarf nach institutionsübergreifender Vernetzung und klaren Standardprozeduren, z. B. im Umgang mit Kindeswohlgefährdung.

## Abstract (en)

**Background:**

Mentally ill parents pose a risk factor for the transgenerational transmission of mental disorders. Contemporary psychiatry mainly focuses on the individual and patients are not always recognized as being parents.

**Objective:**

The development and evaluation of a training program for mental health professionals to support a family orientation in psychiatric treatment by using a bifocal perspective, which keeps an eye on the index patient and the family with a focus especially on children, are presented.

**Methods:**

In order to establish the bifocal perspective in attitudes, knowledge and skills, a half-day training program, consisting of a lecture and a seminar was developed. This was carried out as part of the Children of Mentally Ill Parents –Research Network (CHIMPS-NET) consortium at seven locations in Germany in the respective adult, pediatric and adolescent departments of psychiatry. The needs were evaluated before the training using an online questionnaire. The implementation was accompanied by a qualitative analysis of memory protocols of the trainers.

**Results and discussion:**

The training could be successfully carried out with 120 participants, whereby the target of a comprehensive training of the complete personnel across occupational groups could not be realized, also partly due to the pandemic, and the exercising elements of training could not all be utilized. The evaluation of the questionnaire, which was completed by approximately 50% of the participants showed that the group of participating psychologists and physicians already had a strong family orientation. The qualitative analysis of protocols from all locations documented a strong need for networking across institutions and clear standard procedures, e.g. in dealing with child maltreatment.

Psychisch erkrankte Eltern und ihre Familien zu unterstützen, dient im Sinne einer selektiven Prävention der Unterbrechung der transgenerationalen Weitergabe psychischer Störungen. Gleichzeitig kann die Stärkung der Elternfunktion psychisch kranker Eltern im Sinne einer indizierten Prävention wirksam werden (Lenz und Wiegand-Grefe 2017). Diesen Bedarf, sowohl der betroffenen Kinder als auch der Eltern zugrunde legend, wird eine bifokale Perspektive als Familienorientierung in der psychiatrischen Versorgung, die die Interessen der Eltern, der Kinder und anderer Familienangehörigen berücksichtigen soll, vorgeschlagen. In Form eines Fachkräftetrainings soll die bifokale Perspektive im Rahmen des Konsortiums Children of Mentally Ill Parents – Research-Network (CHIMPS-NET) umgesetzt werden.

## Hintergrund

In Deutschland machen jährlich etwa 175.000 Kinder die Erfahrung, dass ein Elternteil stationär psychiatrisch behandelt wird. Umgekehrt haben ca. 30 % der in Psychiatrien und 60–70 % der in Psychosomatiken behandelten Patient*innen minderjährige Kinder (Mattejat 2014). Studien zeigen, dass Kinder mit psychisch kranken Eltern ein vielfach erhöhtes Risiko tragen, ebenfalls psychisch zu erkranken (Vostanis et al. 2006). Die Risiken einer transgenerationalen Weitergabe psychischer Erkrankungen werden durch eine potenziell niedrige Sensitivität in der Mutter-Kind-Interaktion (Kluczniok et al. 2016; Muzik et al. 2017), einen verstärkten elterlichen Rückzug (Sethna et al. 2017) und ein erhöhtes Risiko für Kindesmisshandlung (Takehara et al. 2017) erklärt. Gleichzeitig schätzen psychisch erkrankte Eltern die psychischen und Verhaltensprobleme bei ihren Kindern höher ein und äußern ein stärkeres Bedürfnis nach Unterstützung in ihrer Elternrolle, verglichen mit einer gesunden Kontrollgruppe (Markwort et al. 2016).

## Implementierungsstudie „Einstellungen, Wissen und Fertigkeiten der Fachkräfte“ im CHIMPS-NET-Konsortium

### Projekt, Ziele und Konzeption

Das CHIMPS-NET-Konsortium ist ein dreijähriges Projekt aus dem Bereich neuer Versorgungsformen des Innovationsfonds des Gemeinsamen Bundesausschusses und hat das Ziel, 4 neue Versorgungsformen für Kinder und Jugendliche (0 bis 21 Jahre) mit psychisch kranken Eltern an 20 Standorten in 14 Bundesländern zu implementieren und zu evaluieren. Die 4 Versorgungsformen werden nach Indikationsstellung passgenau eingesetzt und reichen je nach gesundheitlicher Ausgangslage von Prävention über Multifamilientherapie bis hin zu familienorientierter Therapie sowie einer Online-Therapie. Das Konzept der Interventionen orientiert sich an den individuellen Bedarfen der Familien und integriert psychoanalytische Familientherapie mit psychoedukativen und systemischen Elementen (ausführlich bei Wiegand-Grefe et al. 2011). Alle im CHIMPS-NET realisierten Versorgungsformen zielen darauf ab, die psychische Gesundheit und Lebensqualität der Familien zu verbessern. Dazu sollen Verbesserungen der Krankheitsbewältigung, der familiären Kommunikation und Beziehungen sowie die Überwindung sozialer Isolation und weiterführende Hilfen angestoßen werden.

Die Wirksamkeit der verschiedenen Versorgungsformen wird in randomisierten klinischen Studie („randomized controlled trials“, RCT) untersucht; die Details zu den Evaluationsstudien für die Interventionen finden sich im Studienprotokoll (Wiegand-Grefe et al. in Vorbereitung). Um die Rekrutierungsziele zu erreichen, wurde in das CHIMPS-NET eine übergeordnete randomisierte kontrollierte Implementierungsstudie integriert, die die allgemeine Machbarkeit und Durchführung untersucht. Details dieser Implementierungsstudie werden im Studienprotokoll dieser Studie publiziert (Laser et al. im Druck). In dieser Studie werden in zufällig ausgewählten Kliniken Implementierungsunterstützungen durch 3 weitere Teilprojekte realisiert, während die anderen Kliniken keine derartige Unterstützung erhalten. Diese Teilprojekte befassen sich mit Zugangswegen in die CHIMPS-Interventionen durch externe Zuweiser, Screeninginstrumente zur Identifikation betroffener Familien und ein Training der Klinikfachkräfte zur Stärkung von Einstellungen, Wissen und Fertigkeiten zum Thema Familien mit psychisch kranken Eltern.

Das Teilprojekt zum Training der Fachkräfte wird im Folgenden dargestellt, und erste Ergebnisse werden berichtet. Hierzu werden zunächst Konzept und Inhalte des Trainings vorgestellt. Daran anschließend wird die Evaluation der Trainings dargestellt, die auf den Erfahrungen der Trainerinnen mit dem Training und auf ersten Ergebnissen der begleitenden Studie basiert. Die übergeordnete Fragestellung, ob ein Fachkräftetraining die Zuweisung von Familien in das CHIMPS-NET sowie das Wissen und Fertigkeiten im Hinblick auf die Berücksichtigung der bifokalen Perspektive verbessert, kann an dieser Stelle nicht beantwortet werden. Die gesamte Implementierungsstudie wurde als Studienprotokoll zusammengefasst (Laser et al. im Druck).

Das Training und die Begleitstudie zur Stärkung von Einstellungen, Wissen und Fertigkeiten im Hinblick auf eine Familienorientierung von Fachkräften im Gesundheitswesen basieren auf internationalen Vorstudien. So wurden in den vergangenen Jahren z. B. in Portugal, Norwegen, Irland und Australien verschiedene Bemühungen unternommen, in der Erwachsenenpsychiatrie einen Paradigmenwechsel von einem Patient*innenfokus zu einem Familienfokus mit stärkerer Berücksichtigung der Kinder zu erzielen (Lauritzen et al. 2014; van Doesum et al. 2019). Zu diesem Zweck implementierten Lauritzen et al. (2014) 2 Interventionen: die Einführung eines Erhebungsinstrumentes zur routinierten Erfassung von Familieninformationen sowie den Einsatz von Familiengesprächen. Die Effekte der Maßnahmen wurden longitudinal nach 3 und nach 5 Jahren erfasst, mit dem Ergebnis, dass die Zahl identifizierter Eltern-Patient*innen über den Verlauf deutlich zunahm. Das Wissen der Klinikmitarbeitenden über die Familienorientierung im Gesundheitswesen wuchs, während positive Einstellungen bezüglich der Familienorientierung zunächst zunahmen (Lauritzen et al. 2014), in der Follow-up-Analyse aber tendenziell geringer wurden (Lauritzen et al. 2018). Van Doesum et al. (2019) berichten von einer Zunahme von Wissen und Fertigkeiten über familienorientierte Praxis bei Fachkräften der Erwachsenen- sowie der Kinder- und Jugendpsychiatrischen Klinik durch ein Training in familienorientierter Kurzintervention in der Klinikroutine. Das Training bestand aus einer Vorlesung, Rollenspielen sowie Diskussionen zu Hürden und Lösungen einer erfolgreichen Implementierung. Die Ergebnisse dieser Studien weisen einerseits auf die Herausforderungen hin, Implementierungsprozesse erfolgreich und langfristig umzusetzen. Andererseits erscheint der Ansatz vielversprechend, haltungs-, einstellungs- und kompetenzorientierte Fachkräftetrainings zur Förderung von Implementierungsprozessen zu verwenden.

Studienergebnisse aus Norwegen zeigten, dass die Einstellungen von Kliniker*innen im Gesundheitswesen zusammengefasst durch den Slogan „not mine, not trained, too busy, too risky“ gekennzeichnet sind (freie dt. Übersetzung: „nicht meins, nicht fortgebildet, zu beschäftigt, zu riskant“; Lauritzen et al. 2014). In ihrer täglichen Praxis gaben 44 % der Gesundheitsfachkräfte an, dass sie ihre Patient*innen dazu befragen, ob sie Kinder hätten oder nicht (Lauritzen et al. 2015). Dabei schien das Wissen über Kinder psychisch erkrankter Eltern und über gesetzliche Bestimmungen für deren Versorgung einen positiven Einfluss auf die Identifikation zu nehmen. Zudem hatten Fachkräfte, die angaben, mehr Wissen über Kinder psychisch erkrankter Eltern zu haben, weniger Bedenken, die Arbeitsbeziehung zu den Eltern durch das Einbringen eines Familienfokus zu belasten. Bezüglich der Einstellungen zeigte sich außerdem, dass positive Erwartungen zur Wirksamkeit von Familieninterventionen ein wichtiger Prädiktor für positive Einstellungen bezüglich dieser Praxis waren. Die Ergebnisse unterstreichen die Notwendigkeit, Prozesse im Gesundheitswesen anzukurbeln, die zur Implementierung familienorientierter Praxis beitragen und dies über wirksame Methoden anzugehen, die zunächst die Wahrnehmung und Praxis im Alltag tangieren.

Nach dem Wissen der Autoren liegt im deutschsprachigen Raum bislang keine Untersuchung zu Einstellungen von Klinikmitarbeitenden unterschiedlicher Professionen in der Erwachsenen- und Kinder- und Jugendpsychiatrie zum Thema eines familienorientierten Gesundheitswesens vor. Ebenso wenig ist bekannt, wie bewusst die Thematik bereits in den Alltag der Kliniker*innen integriert ist, was sich beispielsweise anhand eines hohen Anteils an Patient*innen darstellen lässt, von denen die Klinker*innen wissen, ob die Patienten Eltern sind oder nicht. Aus diesem Grund verfolgt die vorliegende Studie 2 Ziele: 1) die Beantwortung der Fragestellung, wie stark das Bewusstsein für Eltern-Patient*innen im psychiatrischen Alltag bei Fachkräften ausgeprägt und somit der Bedarf an einem Training ist sowie 2) die qualitative Evaluation der Akzeptanz und Wirksamkeit eines Fachkräftetrainings zur Verbesserung von Einstellungen, Wissen und Fertigkeiten einer familienorientierten Psychiatrie auf Basis der Gedächtnisprotokolle der Trainerinnen.

### Teilprojekt Fachkräftetraining

#### Struktur, Inhalte und Ziele

Ein integratives Literaturreview über das Konzept des familienfokussierten Gesundheitswesens identifizierte u. a. die folgenden Faktoren als relevant: Psychoedukation für die Familie, Erhebung von Familieninformationen und -funktionen, Koordination zwischen unterschiedlichen helfenden Systemen und Kooperation mit allen Familienmitgliedern (Foster et al. 2015). Das vom Institut für Psychosoziale Prävention des Universitätsklinikums Heidelberg entwickelte Training zielt darauf ab, eine bifokale Perspektive bei den medizinischen Fachkräften zu etablieren, d. h., sowohl die psychisch kranken Eltern als auch die Kinder und weitere Familienmitglieder in den professionellen Blick zu nehmen sowie ein klinikenübergreifendes und abgestimmtes Behandlungsangebot zu etablieren. Die Grundidee einer familienorientierten Psychiatrie besteht darin, die Folgen der psychischen Erkrankung für das Familiensystem professionell einzuschätzen, Symptome aus dem familiären Kontext heraus zu verstehen, Hilfsangebote auszusprechen und beim Vorliegen einer Kindeswohlgefährdung auch im Sinne des Kinderschutzes einzuschreiten. Kindeswohlgefährdendes Verhalten umfasst sowohl Gefahren für die psychische und physische Gesundheit von Kindern als auch Belastungen für ihre soziale und emotionale Entwicklung. Nach dem Bundeskinderschutzgesetz des SGB VIII (BKiSchG) besteht inzwischen eine ausreichende rechtliche Grundlage, die es medizinischen Fachkräften ermöglicht einzuschreiten, wenn kein ausreichender Schutz für Kinder im Sinne einer gesunden Entwicklung besteht. Alle Heilberufler*innen können nach § 4 des BKiSchG beim Verdacht auf Kindeswohlgefährdung tätig werden und sind somit von der Geheimnisträgerschaft entlastet. Dazu können sie dies mit den Betroffenen erörtern, auf die Inanspruchnahme von Hilfen hinwirken, sich durch Fachkräfte extern beraten lassen und dürfen das Jugendamt informieren. Auf dieser gesetzlichen Grundlage können Standardverfahren für das Vorgehen („standard operating procedures“, SOP) abgeleitet werden.

Um Kliniker*innen in ihren Einstellungen, ihrem Wissen und ihren Fertigkeiten zu unterstützen, eine familienorientierte Praxis an ihrer Klinik umzusetzen, wurde das Training folgendermaßen konzipiert: Zu vermittelnde Kenntnisse umfassen die transgenerationale Weitergabe psychischer Erkrankung und rechtliche Aspekte der Kindeswohlgefährdung. Die Fertigkeiten umfassen die Themen Haltung, Kommunikation und Perspektiverweiterung in der Kontaktgestaltung mit bifokaler Perspektive auf die Familie.

Das Training war in 2 Teile gegliedert. Der erste Teil bestand aus einer Vorlesung der Erstautorin, über die die oben genannten Kenntnisse vermittelt wurden. Außerdem wurde die bifokale Haltung in einem Beispielvideo zur Familienorientierung und zur Abklärung von Kindeswohlgefährdung im Gespräch mit einem erwachsenen Patienten dargestellt. Der zweite Teil des Trainings wurde als interaktives Seminar geplant. Struktur, Inhalt und Ziele des Seminars können Tab. 1 entnommen werden.

**Table 1:** 

Inhalte	Ziele
*1. Austausch zur Vorlesung* Sammlung von Eindrücken bzw. offenen Fragen zur Vorlesung sowie zu den Interessen der Teilnehmer für das interaktive Seminar	Ermittlung von Schwerpunkten für das interaktionelle Seminar sowie Klärung von grundlegenden Fragen
*2. Bifokale Perspektive: Diskussion und Reflexion des Beispielvideos zur Familienorientierung im Gespräch* Diskussion: Wie wurden die Interventionen von den Teilnehmenden eingeschätzt? Wie wäre deren Herangehensweise? Welche vergleichbaren Situationen kennen sie aus ihrem Arbeitskontext, und wie haben sie dabei gehandelt?	Bewusstwerdung bzw. Entwicklung einer eigenen Haltung zur bifokalen Perspektive sowie die Bewusstwerdung von dazugehörigen therapeutischen Interventionen
*3. Vorgehen bei möglicher Kindeswohlgefährdung* Wiederholung der wichtigsten theoretischen Punkte; Fragen an die Teilnehmenden: Wie wird in der eigenen Klinik damit umgegangen? Gibt es eine SOP? Was würden Sie sich hier wünschen?	Aneignung von Wissen sowie dem praktischen Umgang mit (potenzieller) Kindeswohlgefährdung. Austausch über den Umgang mit (potenzieller) Kindeswohlgefährdung innerhalb der eigenen Klinik
*4. Psychische Erkrankung kindgerecht erklärt* Austausch über die Erfahrungen, ob und wie die Patient*innen den eigenen Kindern ihre psychische Erkrankung erklären. Was wären Möglichkeiten hierzu? Ergänzend wird eine Herangehensweise vorgestellt, die mit kindgerechter Literatur arbeitet	Bereitstellung von Ideen für die Eltern, um mit ihren Kindern über die eigene psychische Erkrankung in Kontakt zu treten
*5. Übungen in Gesprächsführung zur bifokalen Perspektive* Auflistung möglicher therapeutischer Interventionen sowie Austausch über den praktischen Umgang hiermit	Wissen über mögliche therapeutische Interventionen und Schritte, sich einem Perspektivenwechsel annähern zu können
*6. Offene Fragen*	Klärung von offenen Fragen

*SOP* „standard operating procedure“

#### Durchführung

##### Standorte und Format.

Das Training zur Verbesserung der Einstellungen, des Wissens und der Fertigkeiten bezüglich einer familienorientierten Praxis in der Psychiatrie wurde an 8 Standorten und 16 Kliniken angeboten. Je Standort sollte potenziell eine Erwachsenen- und eine Kinder- und Jugendpsychiatrie teilnehmen. Die Standorte sind Teil des CHIMPS-NET-Konsortiums, wurden aus 20 Standorten des gesamten Konsortiums zufällig randomisiert und liegen über ganz Deutschland verteilt. Aufgrund der durch die „coronavirus disease 2019“ (COVID-19) ausgelösten Pandemie musste das Format des ursprünglich als Präsenzveranstaltung geplanten Trainings angepasst werden. Um die Inhalte möglichst vielen Mitarbeitenden zugänglich zu machen, wurde das Training 2‑teilig gestaltet: Im ersten Teil wurden die inhaltlichen Grundlagen mithilfe einer aufgezeichneten Videopräsentation vermittelt; die Präsentation wurde den Standorten dauerhaft zur Verfügung gestellt. Im zweiten Teil, der aufgrund der Pandemie als Webinar durchgeführt wurde, wurden die vertiefte Bearbeitung der Inhalte angeregt sowie der Austausch über die Inhalte, Verbesserungspotenziale der Standorte und bereits bestehende Netzwerke. Die Video-Aufzeichnung sowie das interaktive Seminar dauerten jeweils eineinhalb Stunden. Das Training und die Begleitstudie wurden durch die jeweiligen Klinikleitungen unterstützt und über Koordinator*innen vor Ort organisatorisch umgesetzt. Pro Standort wurden spezifische technische Hürden im Kontakt mit den Koordinator*innen gelöst (z. B. keine technische Ausstattung am Arbeitsplatz oder fehlende Freischaltungen für Videokonferenzen; aufgrund der COVID-19-Sicherheitsmaßnahmen begrenzte Möglichkeit, die zur Verfügung stehenden Hörsäle für eine gemeinsame Teilnahme am Webinar zu nutzen). Das Training wurde mit Flyern beworben, um eine Teilnahme über alle Professionen, die therapeutisch/pflegerisch mit Patient*innen arbeiten, hinweg in den psychiatrischen Kliniken zu erreichen. Die Trainings wurden flexibel zu Uhrzeiten und an Tagen durchgeführt, die von den Koordinator*innen vorgeschlagen wurden; die Teilnehmer*innen konnten während ihrer Arbeitszeit an der für sie kostenlosen Fortbildung teilnehmen.

Die Trainings fanden ab Oktober 2020 statt, wobei die Videovorlesung den Mitarbeitenden eine bis 2 Wochen vor dem interaktiven Webinar von den Kliniken zur Verfügung gestellt bzw. diese in Klinikhörsälen ausgestrahlt wurde. Um die Ziele des Austausches unter möglichst vielen Mitarbeitenden zu erreichen, wurde das Webinar mehrmals angeboten. Die übergeordnete Implementierungsstudie wurde von der Ethik-Kommission der Ärztekammer Hamburg bewilligt; die jeweiligen Standorte holten zusätzliche Ethikvoten vor Ort ein. An 7 von den angesprochenen 8 Standorten fanden insgesamt 9 Trainings statt; ein Standort schied aus der Studie aufgrund eines negativen lokalen Ethikvotums aus.

##### Methoden.

An allen 7 Standorten wurden eine strukturierte teilnehmende Beobachtung sowie eine Vor- und Nachuntersuchung (unmittelbar vor dem ersten Trainingsteil und nach 28 Tagen) über die Online Plattform SoSci Survey durchgeführt. Einmalig wurden soziodemografische Informationen (u. a. Alter, Geschlecht, Dauer der Berufstätigkeit und berufliche Qualifikation) erfasst. Für die hier vorgestellte Auswertung werden nur die soziodemografischen Information sowie ein selbst generiertes Item berichtet, das die Anzahl der identifizierten Elternpatient*innen erfasst: „Von wie vielen Ihrer Patient*innen (in Prozent) wissen Sie, ob sie Kinder haben“? erhoben. Die Antworten wurden auf einer visuellen Analogskala von 0 bis 100 % erfasst.

##### Strukturierte teilnehmende Beobachtung.

Zur stetigen Evaluation und Adaptation wurden direkt im Anschluss an die Webinare ausführliche Protokolle von den Trainingsleiterinnen (L.K. und S.H.) erstellt, die auch dem Austausch mit der Konsortialführung und Mitarbeitenden der anderen Implementierungsprojekte dienten. Insgesamt wurden 9 Protokolle ausgewertet. Die Protokolle bezogen sich auf Beobachtungen der Trainingsleiterinnen sowie auf Aussagen der Teilnehmenden während des interaktiven Seminars.

### Stichprobe

Über die Zahl der Teilnehmenden für die Vorlesung und damit den ersten Teil des Trainings lässt sich keine Aussage treffen, da die Vorlesung im Videoformat frei zur Verfügung gestellt wurde. Am zweiten Teil des Trainings nahmen 120 Personen teil, wobei die Teilnehmendenzahl zwischen den 7 Standorten von 3 bis 45 Personen schwankte, mit einem Mittel von 13 Personen/Trainingsdurchführung. Entgegen der Planung gelang es nur bei 2 Trainings, dass sowohl Mitarbeitende der Erwachsenen- als auch der Kinder- und Jugendpsychiatrie vertreten waren. Eines der Trainings erfolgte ausschließlich mit Fachkräften einer Kinder- und Jugendpsychiatrie, und in den restlichen 6 Trainings waren Teilnehmende ausschließlich aus der Erwachsenenpsychiatrie vertreten.

An der begleitenden Fragebogenstudie nahmen 58 Fachkräfte aus den 7 Standorten teil (ca. 50 % der Teilnehmenden der interaktiven Webinare). Aufgrund der anonymen Erhebung kann nicht nachvollzogen werden, wie sich die Teilnehmenden auf die Standorte verteilen. Die Beschreibung der Stichprobe ist Tab. 2 zu entnehmen. Im Durchschnitt waren die Teilnehmenden an der begleitenden Studie M = 38,16 Jahre alt (SD ± 9,47 Jahre) und wiesen eine Berufserfahrung von durchschnittlichen M = 9,4 Jahren (SD ± 8,9 Jahre) auf. Der Schwerpunkt der klinischen Tätigkeit lag vermehrt im kinder- und jugendpsychiatrischen Bereich (48,2 %), während 12,5 % der Teilnehmenden angaben, sowohl im Erwachsenen- als auch im kinder- und jugendpsychiatrischen Bereich tätig zu sein. Mit 50 % war die Fachgruppe der Ärzt*innen am stärksten vertreten. Von den Teilnehmenden gaben 44,6 % an, bereits an einer Fortbildung zum Thema psychische Erkrankungen in der Familie teilgenommen zu haben (Tab. 2).

**Table 2:** 

Variable	Verteilung
Alter (Jahre; M ± SD)	38,16 ± 9,47
Ausübung des aktuelles Berufs (Jahre; M ± SD)	9,36 ± 8,98
Geschlecht (Anteil, %)	
– Weiblich	67,2
– Männlich	32,8
– Divers	0,0
Schwerpunkt (Anteil, %)	
– Kinder und Jugendliche	48,2
– Erwachsene	39,3
– Beides	12,5
Qualifikationen^a^ (Anteil, %)	
– Fach- oder Assistenz*ärztin	50,0
– Psycholog*in	20,7
– Psychologische*r Psychotherapeut*in oder Kinder- und Jugendpsychotherapeut*in	15,5
– (Sozial‑)Pädagog*in	15,5
– Praktikant*in/Auszubildende/PiA	8,6
– Ergo‑/Musik‑/Physio‑/Kunsttherapeut*in	3,4
– Teilnahme an Trainings zu psychischen Erkrankungen in der Familie	44,6

*PiA* Psychotherapeut*in in Ausbildung

^a^Für dieses Item waren Mehrfachantworten möglich

### Auswertungsmethoden

#### Deskriptive Statistiken

Um die Fragen zu beantworten, wie vor der Trainingsteilnahme die Einstellungen bezüglich Weiterbildungen im familienorientierten Gesundheitswesen ausfielen und wie hoch die Rate von als Eltern identifizierten Patient*innen war, werden deskriptive Statistiken dargestellt. Die Analysen wurden mit IBM SPSS durchgeführt.

#### Qualitative Inhaltsanalyse

Für die qualitative Auswertung wurden die Gedächtnisprotokolle des interaktiven Webinars herangezogen. Die Protokolle wurden von den Trainingsleiterinnen direkt im Anschluss an das Webinar verfasst. Die Protokolle beinhalteten im ersten Schritt allgemeine Eindrücke und Rückmeldungen der Teilnehmenden zum ersten Teil des Trainings, und im zweiten Teil dokumentieren sie die allgemeinen Eindrücke der Trainingsleiterinnen in Bezug auf das interaktive Webinar. Letztere beziehen sich auf Beobachtungen, inwiefern beispielsweise die Zahl der Teilnehmenden, das Verhältnis der Teilnehmenden aus Erwachsenen- und Kinder- und Jugendpsychiatrie sowie die Anwesenheit der Klinikleitung, aber auch die technische Umsetzung des Webinars potenziell Einfluss auf den Austausch gehabt haben könnten. Im letzten Abschnitt wurde der Interessenschwerpunkt der Teilnehmenden erfasst.

Für die Auswertung der Gedächtnisprotokolle wurde eine inhaltlich-strukturierende qualitative Inhaltsanalyse (Mayring 2004) genutzt. Die Analyse basierte auf 9 Gedächtnisprotokollen von 7 Standorten. An 2 Standorten erfolgte das Training zu 2 Zeitpunkten. Innerhalb der Protokolle und über die Seminare hinweg wurden in einem systematischen Reduktionsprozess Kategorien gebildet, wobei die Häufigkeiten der Kategorien ausgewertet wurden (s. unten). Die Häufigkeiten beziehen sich auf die Anzahl der Nennungen pro durchgeführtem Training und werden hinter den extrahierten Aussagen in Klammern aufgeführt.

### Ergebnisse

#### Quantitative Erhebung

Im Mittel gaben die Teilnehmenden an, von 73,65 % ihrer Patient*innen zu wissen, ob sie Eltern sind oder nicht. Diese Einschätzung variierte jedoch, und 17,1 % gaben an, über diese Information nur bei 50 % oder weniger ihrer Patient*innen zu verfügen. Dagegen teilten 34,5 % der Befragten mit, diese Information von 100 % ihrer Patient*innen erhalten zu haben. Hierbei muss berücksichtigt werden, dass über die Hälfte der Teilnehmenden an der Online-Umfrage im Bereich der Kinder- und Jugendpsychiatrie tätig sind.

#### Qualitative Evaluation^1^

##### Aussagen zur Vorlesung.

Bei einem überwiegenden Teil der Standorte wurde die Vorlesung auf den Klinikservern hinterlegt (4), jedoch gab es teils Übertragungsschwierigkeiten (3). Nicht alle Teilnehmenden hatten zuvor die Vorlesung angesehen (3). Die Vorlesung wurde als gute Zusammenfassung und Übersicht zum Thema bifokale Perspektive aufgefasst (3), wobei die Inhalte teilweise bereits bekannt waren (2). Besonderes Interesse galt dem Thema Kindeswohlgefährdung und den damit einhergehenden Anzeichen (3); als weniger relevant wurden die vorgestellten Gesprächstechniken angesehen (3). Zudem wurde kritisch angemerkt, inwiefern andere Berufsgruppen das Konzept der bifokalen Perspektive umsetzen können (1).

##### Aussagen zum interaktiven Webinar.

Die technische Umsetzung des interaktiven Webinars erfolgte in 6 Durchgängen zufriedenstellend, in 3 Durchgängen war die Interaktion durch die unzureichende Umsetzung (kein Bild oder Ton der Teilnehmenden) deutlich eingeschränkt. In der Mehrheit der Durchgänge nahmen die Teilnehmenden an ihrem Arbeitsplatz (5) oder gemeinsam im Hörsaal (3) und nur vereinzelt aus dem Homeoffice (2) teil, es waren auch Kombinationen davon möglich. Bei 4 Durchgängen waren die Klinikleitenden vertreten, und bei 2 Durchgängen nahmen Mitarbeitende aus der Erwachsenenpsychiatrie und der Kinder- und Jugendpsychiatrie teil. Ein Durchgang erfolgte ausschließlich an einer Kinder- und Jugendpsychiatrie. Die Teilnehmenden waren größtenteils Ärzt*innen, Psycholog*innen oder Psychotherapeut*innen. Nur bei 2 Durchgängen waren auch andere Berufsgruppen vertreten; bei 2 dieser Durchgänge waren dies Sozialarbeiter*innen. Insgesamt war die Beteiligung der Teilnehmenden zufriedenstellend: Zu 3 Durchgängen wurde explizit eine gute Beteiligung vermerkt. Jedoch war die Beteiligung bei 2 Durchgängen deutlich zurückhaltend; hier wurde vermutet, dass dies mit der Anwesenheit der Klinikleitung einherging. In 3 Durchgängen wurde die Expertise der Klinik zum Thema Familienfokus bereits zu Anfang von den Teilnehmenden stark betont. Innerhalb des Seminars wurden besonders das Standardvorgehen bei Anzeichen zur Kindeswohlgefährdung vorgestellt und besprochen (6), Kooperationen besprochen und geknüpft sowie bestehende Projekte benannt (5).

## Diskussion

Ziele der vorliegenden Studie waren die Entwicklung und die Evaluation eines Trainings zur Verbesserung der Einstellung, des Wissens und der Fertigkeiten von medizinischen Fachkräften im Hinblick auf eine Familienorientierung in der Psychiatrie. Zunächst kann resümiert werden, dass ein Training entwickelt werden konnte, das auch unter Coronabedingungen im digitalen Format durchführbar ist und in den klinischen Alltag von Psychiatrien integriert werden kann. Das Training umfasst die international vorgeschlagenen Elemente von Psychoedukation und Koordination der Helfersysteme (Foster et al. 2015) und ergänzt diese um deutschlandspezifische rechtliche Rahmenbedingungen des Kinderschutzes und die Benennung einer therapeutischen familienorientierten Haltung, die als bifokale Perspektive bezeichnet wurde. Trotz der Niedrigschwelligkeit des Trainings (kurze Dauer, kostenlose Teilnahme, Absolvierung während der Arbeitszeit, Klinik bestimmt die Termine) ist es nicht gelungen, den überwiegenden Teil der Gesundheitsfachkräfte der jeweiligen Standorte professionsübergreifend zu schulen. An den interaktiven Webinaren nahmen überwiegend Ärzt*innen und Psycholog*innen teil, was sich auch in der Fragebogenerhebung widerspiegelt. So kann die Studie keine Aussagen zu Akzeptanz und Wirksamkeit im Bereich der Sozialarbeit, der nichtpsychologischen Therapien und der Pflege treffen. Auch ist es zumeist nicht gelungen, dass mehrere Kliniken (Erwachsenen- und Kinder- und Jugendpsychiatrie) gleichzeitig an den Trainings teilnahmen. Die Gründe für die relativ einseitige Teilnahme der akademischen Professionen können an Rekrutierungsmissverständnissen liegen oder im Fallführungsverständnis der Klinikhierarchien begründet sein. Weitere Gründe könnten in der Art des Trainings bestehen (zu digital oder zu sehr auf psychotherapeutisches Arbeiten fokussiert), sodass sich andere Professionen nicht ausreichend angesprochen fühlten. Zukünftige Studien/Trainings sollten daher berücksichtigen, dass diese Zielgruppen vermutlich eine gesonderte Ansprache und vermutlich ein adaptiertes Programm erfordern.

Die weitere Diskussion der Ergebnisse erfolgt also mit der Limitation, dass nur die akademischen medizinischen Fachkräfte repräsentiert sind. Eine weitere Limitation, die bei der Auswertung in Betracht gezogen werden muss, ist, dass von einer selektiven hochmotivierten Stichprobe auszugehen ist, da 44 % der an der Fragebogenstudie Teilnehmenden bereits vor dem Training an Schulungen zur familienorientierten Arbeit teilgenommen hatten. Vor diesem Hintergrund zeigt sich eine Stichprobe von Ärzt*innen und Psycholog*innen, die durchschnittlich von drei Vierteln ihrer Patient*innen wissen, ob diese Kinder haben. Der Wert ist höher als in vergleichbaren Studien bei Gesundheitsfachkräften (Lauritzen et al. 2015) und könnte auf eine stärkere Integration familienorientierter Praxis in den Kliniken hinweisen, die sich im CHIMPS-NET engagieren. Dieser hohe Wert relativiert sich allerdings auch daran, dass über 60 % der Teilnehmenden kinder- und jugendpsychiatrisch tätig sind und somit eher aufgrund des Fokus auf die therapeutische Arbeit mit den Kindern und Jugendlichen in einem Familienfokus arbeiten. Zusammenfassend bestand in der Stichprobe ein hohes Bewusstsein für Eltern-Patient*innen im psychiatrischen Alltag der Fachkräfte, womit sehr gute Bedingungen für die Durchführung eines Fachkräftetrainings zur Familienorientierung gegeben sind. Andererseits zeigen die Ergebnisse auf, dass viele Patient*innen nicht als Eltern identifiziert werden, was möglicherweise durch die Vermittlung von Wissen über Kinder psychisch erkrankter Eltern, gesetzliche Bestimmungen und Behandlungsmöglichkeiten sowie eine Förderung positiver Einstellung und Wirksamkeitserwartungen und den Abbau von Bedenken gesteigert werden kann (Foster et al. 2015; Lauritzen et al. 2015). Es bleibt zu untersuchen, ob eine solche Fortbildung auch die gewünschten Effekte erzielen kann. Die qualitative Evaluation der Akzeptanz und Wirksamkeit des tatsächlichen Fachkräftetrainings spiegelte diesen Eindruck ebenfalls wider. In Bezug auf die Wissensvermittlung bestand bereits ein hohes Grundlagenwissen bei den Teilnehmenden; lediglich im Bereich der rechtlichen Grundlagen konnte das Training substanziell Neues und im Hinblick auf die Symptome von Kindeswohlgefährdung beitragen. In dem digitalen Format gelang es jedoch weniger gut, übende Elemente für eine verbesserte oder motivierende Gesprächsführung umzusetzen. Hier wäre also ein „Face-to-face“-Element die Voraussetzung für eine gelingende Schulung, sodass eine Umsetzung in der konkreten Praxis mithilfe von Rollenspielen an Musterfällen simuliert werden könnte. Besonders bedeutsam erschienen den Teilnehmenden die Netzwerkarbeit und eine klare Übereinkunft am jeweiligen Standort über Verantwortlichkeiten und Vorgehensweisen im Sinne einer SOP, d. h., dass ein Training an der Stelle als nachhaltig erlebt wird, wenn es strukturenbildend oder -klärend ist. Hier zeigten sich jedoch auch die Grenzen einer institutionsüberschreitenden gemeinsamen SOP zwischen Erwachsenen- und Kinder/Jugendpsychiatrie, die zu etablieren den Rahmen eines Trainings überschreitet. In den Webinaren wurde deutlich, dass der Austausch insbesondere in den Standorten gut gelang, in denen bereits in der Vergangenheit gemeinsam im Sinne einer Familienorientierung klinikübergreifend gearbeitet wurde. Ein einmaliges Training kann diese Form der kontinuierlichen institutionellen Zusammenarbeit nicht ersetzen, sondern nur weiteren Rückhalt bieten oder Anstoß dazu geben. Da die meisten Standorte bereits Programme zur familienorientierten Arbeit implementiert hatten (CHIMPS und andere), ist auch auf der Ebene der Institution von einer gewissen Selektion auszugehen und die Stichprobe an teilnehmenden Kliniken kann daher nicht als repräsentativ angesehen werden. Gleichwohl verweisen diese Kliniken auf einen möglichen Paradigmenwechsel in der Psychiatrie in Richtung einer Familienorientierung.

## Fazit für die Praxis

- Eine Familienorientierung in der Psychiatrie setzt das Einnehmen einer bifokalen Perspektive voraus.
- Psychische Erkrankungen beeinflussen nicht nur das Individuum und entstehen nicht nur im Individuum. Daher setzt eine familienorientierte Psychiatrie sowohl bei den Indexpatient*innen als auch bei den Familien an und versucht, beide Perspektiven im Blick zu halten.
- Dazu sind neue Standardprozeduren und eine institutionenübergreifende Vernetzung sinnvoll, die durch Fachkräfteschulungen sinnvoll unterstützt werden können.

## Footnotes

1 Angaben in Klammern beziehen sich auf die Anzahl der Nennungen pro durchgeführtem Training.

## References

[CR17] van Doesum K, Maia T, Pereira C, Loureiro M, Marau J, Toscano L, Lauritzen C, Reedtz C (2019). The impact of the “Semente” program on the family-focused practice of mental health professionals in Portugal. Front Psychiatry.

[CR2] Foster K, McCloughen A, Delgado C, Kefalas C, Harkness E (2015). Emotional intelligence education in pre-registration nursing programmes: an integrative review. Nurse Educ Today.

[CR4] Kluczniok D, Boedeker K, Fuchs A, Hindi Attar C, Fydrich T, Fuehrer D, Dittrich K, Reck C, Winter S, Heinz A, Herpertz SC, Brunner R, Bermpohl F (2016). Emotional availability in mother-child interaction: the effects of maternal depression in remission and additional history of childhood abuse. Depress Anxiety.

[CR5] Laser C, Branzke A, Skogøy BE, Reupert A, Daubmann A, Zapf A, Pawils S, Taubner S, Winter S, Maybery D, Wiegard-Grefe S (submitted) Clinical implementation and evaluation of three implementation interventions for a family-oriented care for children of mentally ill parents (ci-CHIMPS): study protocol for a randomizes controlled multicentre trial 10.3389/fpsyt.2022.823186 PMC8919324 35295776

[CR8] Lauritzen C, Reedtz C, Van Doesum KT, Martinussen M (2014). Implementing new routines in adult mental health care to identify and support children of mentally ill parents. BMC Health Serv Res.

[CR7] Lauritzen C, Reedtz C, Van Doesum K (2015). Factors that may facilitate or hinder a family-focus in the treatment of parents with a mental illness. J Child Fam Stud.

[CR6] Lauritzen C, Reedtz C, Rognmo K, Nilsen MA, Walstad A (2018). Identification of and support for children of mentally ill parents: a 5 year follow-up study of adult mental health services. Front Psychiatry.

[CR9] Lenz A, Wiegand-Grefe S (2017). Kinder psychisch kranker Eltern.

[CR10] Markwort I, Schmitz-Buhl M, Christiansen H, Gouzoulis-Mayfrank E (2016). Psychisch kranke Eltern in stationärer Behandlung. Psychiat Prax.

[CR11] Mattejat F, Mattejat F, Lisofsky B (2014). Kinder mit psychisch kranken Eltern. Nicht von schlechten Eltern. Kinder psychisch kranker Eltern.

[CR13] Mayring P, Flick U, von Kardorff E, Steinke I (2004). Qualitative content analysis. A companion to qualitative research.

[CR14] Muzik M, Morelen D, Hruschak J, Rosenblum KL, Bocknek E, Beeghly M (2017). Psychopathology and parenting: an examination of perceived and observed parenting in mothers with depression and PTSD. J Affect Disord.

[CR15] Sethna V, Perry E, Domoney J, Iles J, Psychogiou L, Rowbotham NEL, Stein A, Murray L, Ramchandani PG (2017). Father-child interactions at 3 months and 24 months: contributions to children’s cognitive development at 24 months. Infant Ment Health J.

[CR16] Takehara K, Suto M, Kakee N, Tachibana Y, Mori R (2017). Prenatal and early postnatal depression and child maltreatment among Japanese fathers. Child Abus Negl.

[CR18] Vostanis P, Graves A, Meltzer H, Goodman R, Jenkins R, Brugha T (2006). Relationship between parental psychopathology, parenting strategies and child mental health. Soc Psychiatry Psychiatr Epidemiol.

[CR19] Wiegand-Grefe S, Halverscheid S, Plass A (2011). Kinder und ihre psychisch kranken Eltern: familienorientierte Prävention-der CHIMPs-Beratungsansatz.

[CR20] Wiegand-Grefe S, Taczkowski J, Branzke A, Leidger A, Adema B, Meyer AK, Hot A, Daubmann A, Zapf A, Kilian R, Becker T, Gallinat J, Karow A, Zeidler J, von der Schulenburg M, Dirmaier J, Pawils S, Taubner S, Willms G, Goerres B, Decarli J, Sekler J, Bender S, Jessen F, Siniatchkin M, Drießen M, Kölch M, Winter SM, Bermpohl F, Heinz A, Flechtner H, Frodl T, Englert E, Schlößer R, Becker K, Kircher T, Reif A, Leibing E, Reich G, Kis B, Fleischhaker C, Domschke K, Noterdaeme M, Schmauß M, Schulte-Körne, Weber B, Renner T, Fallgatter A, Möhler E, In-Albon T, Brünger M, Claus S, Heinze M, Burkhard R, Klein F, Kronmüller KT, Holtmann M, Rummel-Kluge C, Haase C, Brooks A, Born S, Baumeister H (in prep) Evaluation of four tailored, need-adapted interventions for Children and adolescents of mentally ill parens—research network (CHIMPS-NET): Study protocol for a randomized controlled multicenter trial

